# The association between the CUN-BAE index and depression mediated by SIRI: A large cross-sectional study

**DOI:** 10.1371/journal.pone.0334655

**Published:** 2025-10-31

**Authors:** Muxi Li, Yazhi Qi, Mengru Cao, Hongjun Lou, Wei Teng, Long Wang

**Affiliations:** 1 First Clinical Medical College, Heilongjiang University of Chinese Medicine, Harbin, China; 2 Basic Medical College, Heilongjiang University of Chinese Medicine, Harbin, China; 3 The Third Affiliated Hospital of Harbin Medical University, Harbin, China; 4 The First Affiliated Hospital of Heilongjiang University of Chinese Medicine, Harbin, China; Henan Polytechnic University, CHINA

## Abstract

**Background:**

Depression is a common global mood disorder problem and one of the major disabling factors. The Clínica Universidad de Navarra-Body Adiposity Estimator (CUN-BAE) index is an important composite indicator for measuring fat content in the body. Our goal is to find out the relationship between the CUN-BAE index and depression, as well as the mediating effect of the Systemic Inflammatory Response Index (SIRI) between the two.

**Methods:**

All participants aged ≥ 20 from the 2005–2020 cycles of the NHANES in the US were included in the research. To assess the relationships between CUN-BAE and SIRI and depression, we used weighted multivariable logistic regression models. Potential nonlinearity was examined using restricted cubic splines (RCS) regression models. We performed mediation analysis with 1000 bootstrap replicates to evaluate the mediating function of SIRI in the relationship of CUN-BAE and depression.

**Results:**

31,592 participants were examined for the statistical analysis, of whom 28,825 were classified into the non-depression group and 2,767 were identified as belonging to depression group. Individuals in the depression group were more probable to be female and to have diabetes and hypertension, and higher SIRI and CUN-BAE levels. An incremental rise of one unit in CUN-BAE was connected to a 2% elevation in the odds of developing depression (OR = 1.02; 95% CI: 1.01–1.03; *P* < 0.001). The regression analysis revealed a statistically significant non-linear positive relationship between CUN-BAE levels and depressive status (*P* for non-linearity = 0.005), alongside a J-shaped curve characterizing the association between log-transformed SIRI and depression (*P* for non-linearity = 0.007). The AUC of CUN-BAE for predicting depression was 0.60(0.59,0.61). With a 3% mediating effect, SIRI functioned as a partial mediator in the pathway linking CUN-BAE levels to depression.

**Conclusions:**

The level of the CUN-BAE index might serve as a potential index for assessing an individual’s risk of developing depression. Monitoring body fat and inflammation levels might be useful for early detection of those who are potentially at risk for depression.

## 1. Introduction

Due to the accelerated pace of life and changes in the social environment, psychological problems have gradually received extensive attention from society [1]. Depression is a common global mood disorder problem and one of the major disabling factors, with persistent low mood, absence of passion, and delayed thinking as the main clinical features, as well as low recognition, low consultation, and low treatment rate [2]. With the intensification of social competition and the acceleration of urbanization, the prevalence of depression has been rising globally. The prevalence of depression in kids and teenagers has been reported to be as high as 21.3% worldwide, with a constant trend as mentioned above [3]. And 13.3% of older adults suffer from severe depression [4]. Beyond its detrimental impact on individual quality of life, depression imposes considerable burdens on familial dynamics and broader societal functioning. In Austria, the annual direct cost of refractory depression in 2021 is reported to be estimated at €345 million, and the indirect cost is estimated at €684.7 million [5]. However, currently, the treatment of depression is based on oral antidepressants combined with psychotherapy. Antidepressants commonly used in clinical practice usually have a slow onset of action, need to be taken for a long period of time, and have varying degrees of adverse effects, such as nausea, diarrhea, headache, and dry mouth [6,7]. Therefore, the discovery of risk factors for depression and early intervention are of great significance in delaying disease progression and improving patients’ quality of life.

An expanding body of evidence supports a significant relationship between excessive obesity and an increased susceptibility to depression [8], and there are multiple common biological mechanisms between the two, including genetic factors, immune-inflammation, energy metabolism, and gut microbial imbalance [9,10]. Obesity can increase the risk of depression through the interplay of multiple mechanisms, including chronic low-grade inflammation, hypothalamic–pituitary–adrenal (HPA) axis dysregulation, and disturbances in energy metabolism. These processes disrupt the synthesis, release, and metabolism of central neurotransmitters such as serotonin and dopamine, thereby significantly contributing to the development of depression [11,12]. Currently, body mass index (BMI), visceral adiposity index, and other metrics are frequently employed to measure obesity. Although the above indicators can measure the degree of obesity to a certain extent, they are unable to assess the body fat accumulation. The Clínica Universidad de Navarra-Body Adiposity Estimator (CUN-BAE) index, a composite indicator created by Gómez-Ambrosi [13] and others, is comprised of age, gender, and BMI, which can serve as a reliable alternative indicator for measuring body fat percentage. Meanwhile, previous studies have shown that it is more effective in predicting cardiometabolic risk factors with higher correlation than BMI and waist circumference [13]. Therefore, we hypothesize that CUN-BAE may also serve as a potential risk factor for depression; however, this remains to be confirmed by further studies.

Obesity is widely recognized not only as a metabolic disturbance but also as a state characterized by persistent low-grade inflammation. Adipose tissue, especially visceral fat, can release a variety of pro-inflammatory factors, such as interleukin-1β, interleukin-6, monocyte chemotactic protein-1, and so on. In an obese state, this condition stimulates the innate immune system, culminating in a generalized pro-inflammatory state throughout the organism. And depressed patients are often accompanied by increased concentrations of inflammatory signs, such as C-reactive protein and tumor necrosis factor-α. Inflammatory factors can induce or exacerbate depression by interfering with central nervous system function through mechanisms such as affecting neurotransmitter metabolism, down-regulating the level of brain-derived neurotrophic factor, and contributes to the activation of the hypothalamic–pituitary–adrenal axis. The Systemic Inflammatory Response Index (SIRI) is a reliable marker for assessing inflammatory status. Li et al. [14] further demonstrated a significant positive association between SIRI and depression (OR = 1.06, 95% CI = 1.01–1.10, *P* = 0.016). Based on these findings, we hypothesize that SIRI may serve as a potential mediator in the association between CUN-BAE and depression.

Grounded in the aforementioned theoretical framework, this investigation was aiming at elucidating the dose-response relationship between the CUN-BAE index and depression, and to assess the potential mediating effect of the Systemic Inflammatory Response Index (SIRI) in this relationship. The findings are expected to provide a scientific basis for improving earlier detection and preventive strategies for depression.

## 2. Materials and methods

### 2.1. Study design and participants

**Fig 1:**
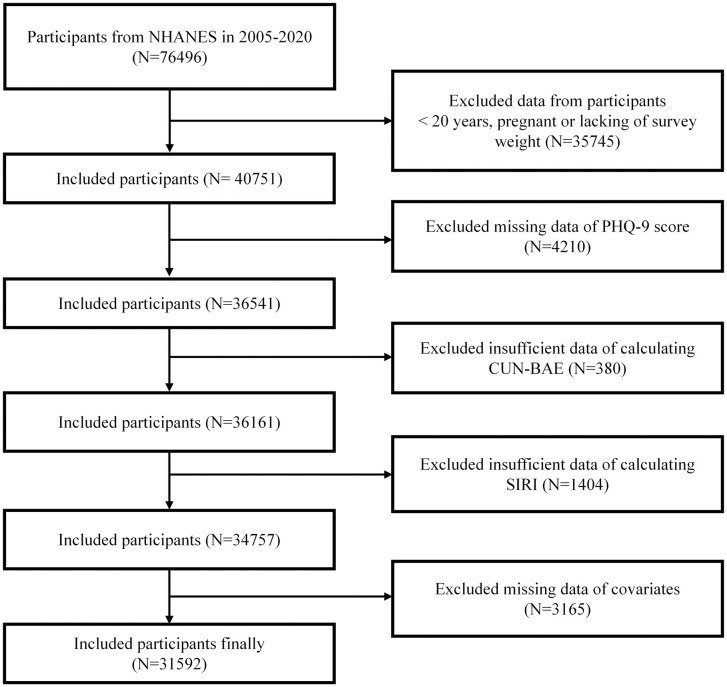
Flow diagram for the study’s participant selection process.

This study includes NHANES participants aged ≥ 20 from the 2005–2020 cycles in the U.S. Other researchers introduced the characteristics of the NHANES database [15]. Fig 1 presents the participant selection process. Out of 76,496 initial respondents, those were excluded if they were younger than 20 years of age, pregnant, or lacked appropriate survey weights (*n* = 35,745); or lack information from Patient Health Questionnaire-9 (PHQ-9) (*n* = 4,210); were missing essential variables for calculating CUN-BAE (*n* = 380) or SIRI (*n* = 1,404); or lacked critical covariate information, including age, sex, or racial classification (*n* = 3,165). Finally, the statistical analysis included 31,592 participants in totality. The protocols of the NHANES survey have got ethical clearance from the National Center for Health Statistics’ Research Ethics Review Board. Everyone who participated signed an informed consent document before starting their involvement.

### 2.2. Definition of depression

Within the context of the present analysis, the PHQ-9 was used to examine the existence of depression. This tool, constructed by Kroenke et al., was applied to quantify the intensity of depressive symptomatology within the last two weeks [16]. The PHQ-9 comprises nine items, each item is rated on a 4-point Likert scale ranging from 0 (“not at all”) to 3 (“nearly every day”), yielding a cumulative score between 0 and 27. Referring to earlier research, depression is described as a total score of PHQ-9 ≥ 10 points [17].

### 2.3. Two indexes and laboratory measurement

All participants underwent interviews at their homes and physical exams at a mobile examination center (MEC). Height and weight data were measured by trained health technicians using standardized protocols at the MEC. The history of diabetes and hypertension was obtained at home through self-reported, collected by trained interviewers using the computer-assisted personal interview system. All participants were instructed to fast for at least nine hours prior to blood collection. The Beckman Coulter DxH 800 instrument was used to perform complete blood count on blood specimens and provide a distribution of blood cells for all participants [18].

The assessment indexes were computed using the following formulas [19,20]:


$$\begin{array}{cc} & CUN-BAE\;=\;-\;44.988\;+\;(3.172\;\times \;BMI)\;+\;(10.689\;\times \;sex)\;+\;(0.503\;\times \;age)\\ & \left(0.026\;\times \;{BMI}^{\text{2}})\;+\;(0.181\;\times \;BMI\;\times \;sex)\;(0.02\;\times \;BMI\;\times \;age)\;+\;(0.00021\;\times \;{BMI}^{\text{2}}\;\times \;age\right)\\ & (0.005\;\times \;{BMI}^{\text{2}}\;\times \;sex).\;Male\;=\;0;\;Female\;=\;1. \end{array}$$

SIRI = (neutrophil count × monocyte count)/lymphocyte count.\]

It is worth emphasizing that both depression and the two indices (CUN-BAE and SIRI) were assessed at the same cross-sectional time point.

### 2.4. Covariates

Covariates including age, gender, race, education level, poverty income ratio (PIR), and marital status were taken from the baseline household questionnaires. Additionally, histories of diabetes and hypertension, drinking and smoking, and other behaviors were gathered. The NHANES website provided the public with further information about the aforementioned covariates.

### 2.5. Statistical analysis

The analysis of the entire study was carried out by R 4.4.1, with two-tailed *P* < 0.05 interpreted as significant. Considering the usage characteristics of the NHANES database, we incorporated MEC examination weights to ensure nationally representative estimates [21]. Baseline variables were contrasted between participants with and without depression. Continuous data were expressed as weighted means with standard deviations and analyzed using weighted t-tests for normally distributed metrics or weighted Wilcoxon rank-sum tests for non-normally distributed parameters. Categorical variables, presented as frequencies and corresponding weighted proportions, were assessed utilizing weighted chi-square tests [22]. Due to the right-skewed distribution of SIRI, SIRI was Ln-transformed prior to analysis [23].

To evaluate the relationship of CUN-BAE and SIRI with depression, weighted multivariable logistic regression models were implemented: Model 1 didn’t include any covariate adjustments, whereas Model 2 was adjusted for demographic variables, including age, sex, race/ethnicity, educational attainment, PIR, and marital status, and Model 3 adjusted for all potential covariates. CUN-BAE and SIRI were both used in continuous analyses and categorical analyses and *P* for trend was calculated. Potential nonlinearity was examined using restricted cubic splines (RCS) regression models [24]. If there is a significant non-monotonic correlation, two-piecewise linear regression based on threshold analysis was employed [25]. Subsequently, subgroup analyses were used to evaluate possible effects of interactions between CUN-BAE and covariates on the risk of depression. The discriminative capacity of CUN-BAE in identifying individuals with depression was evaluated via receiver operating characteristic (ROC) curve analysis [26]. The larger the area under the curve (AUC) value, the higher the predictive performance of CUN-BAE. To assess the intermediary function of SIRI in the relationship between CUN-BAE and depressive outcomes, a mediation analysis was conducted employing 1,000 bootstrap iterations. Mediation analysis included the total effect (TE), direct effect (DE), as well as indirect effect (IE) between variables [27].

## 3. Results

### 3.1. Characteristics of study participants

The research included 31,592 participants, of whom 28,825 were in the non-depression group and 2,767 were in the depression group. Participants in the depression group tended to be female, aged 40–59 years, with lower education levels, lower PIR, current smokers, current moderate drinkers, and had a greater prevalence of diabetes and hypertension than the non-depression group. Importantly, the depression group had significantly higher SIRI and CUN-BAE levels (Table 1).

**Table 1:** Characteristics of participants grouped by depression in NHANES 2005-2020.

Characteristic	Total	Non-depression group	Depression group	*P*
*n*	31,592	28,825	2,767	
Age, *n* (%)				< 0.001
20-39	10,257 (35.4)	9,404 (35.4)	853 (35.1)	
40-59	10,652 (38.2)	9,531 (37.7)	1,121 (43.4)	
≥60	10,683 (26.4)	9,890 (26.9)	793 (21.5)	
Gender, *n* (%)				< 0.001
Male	15,736 (49.1)	14,721 (50.2)	1,015 (36.0)	
Female	15,856 (50.9)	14,104 (49.8)	1,752 (64.0)	
Race, *n* (%)				< 0.001
Mexican American	4,607 (7.9)	4,220 (7.9)	387 (7.3)	
Other Hispanic	2,928 (5.3)	2,581 (5.2)	347 (7.5)	
Non-Hispanic White	13,985 (69.4)	12,788 (69.8)	1,197 (65.5)	
Non-Hispanic Black	6,751 (10.3)	6,131 (10.1)	620 (12.6)	
Other Race – Including Multi-Racial	3,321 (7.1)	3,105 (7.1)	216 (7.0)	
Education, *n* (%)				< 0.001
Less than high school	7,086 (14.3)	6,173 (13.6)	913 (23.2)	
High school grad/GED or equivalent	7,322 (23.5)	6,625 (23.1)	697 (28.3)	
Higher than high school	17,184 (62.2)	16,027 (63.3)	1,157 (48.5)	
PIR, *n* (%)				< 0.001
≤1.3	9,580 (20.0)	8,160 (18.4)	1,420 (39.2)	
1.3-3.5	11,998 (35.7)	1,1046 (35.5)	952 (37.3)	
>3.5	10,014 (44.3)	9,619 (46.0)	395 (23.4)	
Marital status, *n* (%)				< 0.001
Married/living with partner	18,896 (63.9)	17,638 (65.2)	1,258 (49.0)	
Widowed/divorced/separated	7,059 (18.6)	6,130 (17.6)	929 (29.7)	
Never married	5,637 (17.5)	5,057 (17.2)	580 (21.3)	
Smoking status, *n* (%)				< 0.001
Non smokers	17,260 (54.8)	16,140 (56.1)	1,120 (38.3)	
Former smokers	7,832 (25.4)	7,198 (25.6)	634 (23.2)	
Current smokers	6,500 (19.9)	5,487 (18.3)	1,013 (38.5)	
Drinking status, *n* (%)				< 0.001
Non drinkers	9,906 (25.2)	8,925 (24.7)	981 (30.7)	
Current moderate drinkers	19,491 (66.5)	17,956 (67.2)	1,535 (58.8)	
Current heavy drinkers	2,195 (8.3)	1,944 (8.1)	251 (10.5)	
Diabetes, *n* (%)				< 0.001
Yes	4,144 (9.8)	3,593 (9.3)	551 (15.5)	
No	27,448 (90.2)	25,232 (90.7)	2,216 (84.5)	
Hypertension, *n* (%)				< 0.001
Yes	11,569 (32.2)	10,245 (31.2)	1,324 (43.9)	
No	20,023 (67.8)	18,580 (68.8)	1,443 (56.1)	
SIRI, median (25th, 75th)	1.06 (0.73, 1.53)	1.05 (0.73, 1.52)	1.12 (0.75, 1.67)	< 0.001
CUN-BAE, mean (SD)	35.07 (9.91)	34.83 (9.78)	38.05 (10.86)	< 0.001

### 3.2. Association between CUN-BAE, SIRI and depression

Table 2 showed the association between CUN-BAE, SIRI, and depression in weighted logistic regression models. In Model 1, the risk of depression increased by 3% for every unit rise in CUN-BAE (95% CI: 1.03, 1.04, *P* < 0.001). In Model 2, the risk of depression increased by 3% for every unit rise in CUN-BAE (OR: 1.03, 95% CI: 1.02, 1.03, *P* < 0.001). In Model 3, the risk of depression increased by 2% for every unit rise in CUN-BAE (OR: 1.02, 95% CI: 1.01, 1.03, *P* < 0.001). Stratified analysis by CUN-BAE quartiles in Model 3 revealed a statistically significant upward trend in depression risk corresponding to elevated CUN-BAE levels (*P* for trend < 0.001). In contrast to the Q1 group, the Q4 group had a 56% higher risk of depression (OR: 1.56, 95% CI: 1.26, 1.93, *P* < 0.001).

**Table 2:** The associations between CUN-BAE, SIRI and depression in weighted logistic regression models.

	Individuals with depression (*n*)	Total population (*n*)	Model 1		Model 2		Model 3	
			OR (95% CI)	*P*	OR (95% CI)	*P*	OR (95% CI)	*P*
CUN-BAE								
Per 1 unit increment			1.03 (1.03, 1.04)	< 0.001	1.03 (1.02, 1.03)	< 0.001	1.02 (1.01, 1.03)	< 0.001
Q1 (2.45–28.18)	505	7901	Reference		Reference		Reference	
Q2 (28.18–34.85)	514	7895	0.93 (0.76, 1.12)	0.435	1.02 (0.83, 1.24)	0.851	0.99 (0.81, 1.21)	0.908
Q3 (34.85–42.87)	701	7898	1.31 (1.12, 1.53)	0.001	1.26 (1.05, 1.52)	0.012	1.2 (1.0, 1.44)	0.057
Q4 (42.87–59.7)	1047	7898	2.14 (1.84, 2.48)	< 0.001	1.78 (1.44, 2.21)	< 0.001	1.56 (1.26, 1.93)	< 0.001
*P* for trend			< 0.001		< 0.001		< 0.001	
Ln-SIRI								
Per 1 unit increment			1.21 (1.10, 1.33)	< 0.001	1.26 (1.14, 1.39)	< 0.001	1.14 (1.02, 1.26)	0.016
Q1 (0.05–0.69)	652	7922	Reference		Reference		Reference	
Q2 (0.69–1.02)	622	7885	0.91 (0.78, 1.06)	0.22	0.98 (0.83, 1.15)	0.764	0.94 (0.80, 1.11)	0.47
Q3 (1.02–1.50)	696	7993	1.06 (0.91, 1.24)	0.461	1.13 (0.96, 1.34)	0.148	1.05 (0.88, 1.24)	0.587
Q4 (1.50–24.6)	797	7792	1.26 (1.08, 1.46)	0.003	1.33 (1.13, 1.57)	< 0.001	1.15 (0.97, 1.36)	0.109
*P* for trend			< 0.001		< 0.001		0.048	

Model 1 (unadjusted), Model 2 (adjusted for demographics: age, sex, race, education, PIR and marital status), and Model 3 (further adjusted for smoking status, drinking status, diabetes and hypertension).

In Model 1, a one-unit increase in log-transformed SIRI (Ln-SIRI) was related to a 21% elevation in depression risk (OR = 1.21, 95% CI: 1.10–1.33, *P* < 0.001). Model 2 indicated a 26% higher likelihood of depression per unit increase in CUN-BAE (OR = 1.14, 95% CI: 1.03–1.39, *P* < 0.001). In the fully adjusted Model 3, each unit increment in Ln-SIRI corresponded to a 14% rise in depression risk (OR = 1.14, 95% CI: 1.02–1.26, *P* < 0.001). Additionally, quartile-based analysis of SIRI showed a significant trend of increasing depression risk with higher SIRI levels in Model 3 (*P* for trend = 0.048).

### 3.3. Dose-response relationships between CUN-BAE, Ln-SIRI and depression

**Fig 2:**
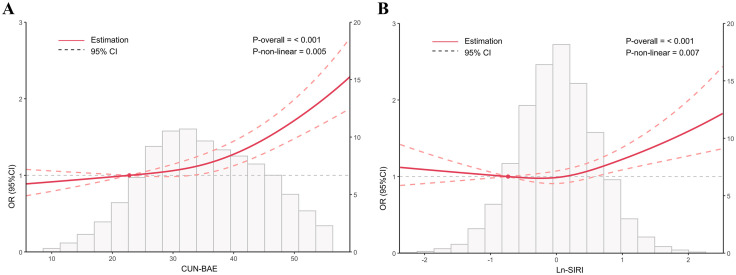
The associations of CUN-BAE and SIRI with depression in RCS regression models. Models were adjusted for age, gender, race, education, PIR, marital status, smoking status, drinking status, diabetes and hypertension.

RCS regression was applied to examine the relationships between CUN-BAE, Ln-SIRI, and depression (Fig 2). The model demonstrated a non-linear positive relationship between CUN-BAE and depression (*P* for non-linear = 0.005) and a J-shaped correlation between Ln-SIRI and depression (*P* for non-linear = 0.007).

Therefore, we further established threshold analysis to investigate the J-shaped correlation between SIRI and depression (Table 3). Threshold effect analysis identified a turning point at Ln-SIRI = −0.364. The log-likelihood ratio test confirmed a significant threshold at Ln-SIRI = 0.364 (*P* = 0.002). When Ln-SIRI was > −0.364, a 22.4% increase in the incidence of depression was linked to every unit rise in Ln-SIRI (OR: 1.224, 95% CI: 1.116, 1.342, *P* < 0.001).

**Table 3:** Two-piecewise logistic regression models of SIRI and depression.

Ln-SIRI	OR (95%CI)	*P*
≤−0.364	0.831 (0.686-1.012)	0.062
>-0.364	1.224 (1.116-1.342)	< 0.001
Log likelihood ratio test		0.002

Models were adjusted for all potential covariates, including age, gender, race, education, PIR, marital status, smoking status, drinking status, diabetes and hypertension.

### 3.4. Subgroup analysis and ROC curve of the predicted performance of CUN-BAE

Stratified analyses across subgroups were conducted to examine the association between CUN-BAE levels and depression (Table 4). The positive correlation between CUN-BAE and depression was consistent in most subgroups. We found that gender, race, marital status, and diabetes regulated associations between CUN-BAE and the risk of depression (*P* for interaction < 0.05). The impact of CUN-BAE on depression is stronger in female, other Hispanic, married/living with partner, and diabetes groups.

**Table 4:** Subgroup analyses.

Characteristic	OR (95%CI)	*P* value	*P* for interaction
Age			0.739
20-39	1.02 (1.01, 1.03)	0.004	
40-59	1.02 (1.01, 1.04)	0.003	
≥60	1.04 (1.02, 1.05)	< 0.001	
Gender			0.047
Male	1.01 (0.99, 1.02)	0.300	
Female	1.03 (1.02, 1.04)	< 0.001	
Race			0.032
Mexican American	1.02 (1.00, 1.04)	0.056	
Other Hispanic	1.05 (1.02, 1.07)	< 0.001	
Non-Hispanic White	1.02 (1.01, 1.03)	0.003	
Non-Hispanic Black	1.00 (0.99, 1.01)	0.413	
Other Race – Including Multi-Racial	1.04 (1.01, 1.07)	0.007	
Education			0.222
Less than high school	1.00 (0.99, 1.02)	0.941	
High school grad/GED or equivalent	1.02 (1.01, 1.04)	0.0056	
Higher than high school	1.03 (1.02, 1.04)	< 0.001	
PIR			0.63
≤1.3	1.02 (1.01, 1.03)	< 0.001	
1.3-3.5	1.02 (1.01, 1.03)	0.004	
>3.5	1.03 (1.01, 1.05)	0.013	
Marital status			0.026
Married/living with partner	1.03 (1.02, 1.04)	< 0.001	
Widowed/divorced/separated	1.01 (1.00, 1.02)	0.157	
Never married	1.01 (1.00, 1.03)	0.07	
Smoking status			0.061
Non smokers	1.04 (1.02, 1.05)	< 0.001	
Former smokers	1.02 (1.00, 1.04)	0.022	
Current smokers	1.00 (0.99, 1.02)	0.502	
Drinking status			0.561
Non drinkers	1.02 (1.01, 1.03)	0.008	
Current moderate drinkers	1.02 (1.01, 1.03)	< 0.001	
Current heavy drinkers	1.02 (1.00, 1.04)	0.078	
Diabetes			0.009
Yes	1.05 (1.03, 1.08)	< 0.001	
No	1.02 (1.01, 1.03)	< 0.001	
Hypertension			0.502
Yes	1.03 (1.01, 1.04)	< 0.001	
No	1.02 (1.01, 1.03)	0.003	

Models were adjusted for age, gender, race, education, PIR, marital status, smoking status, drinking status, diabetes and hypertension.

**Fig 3:**
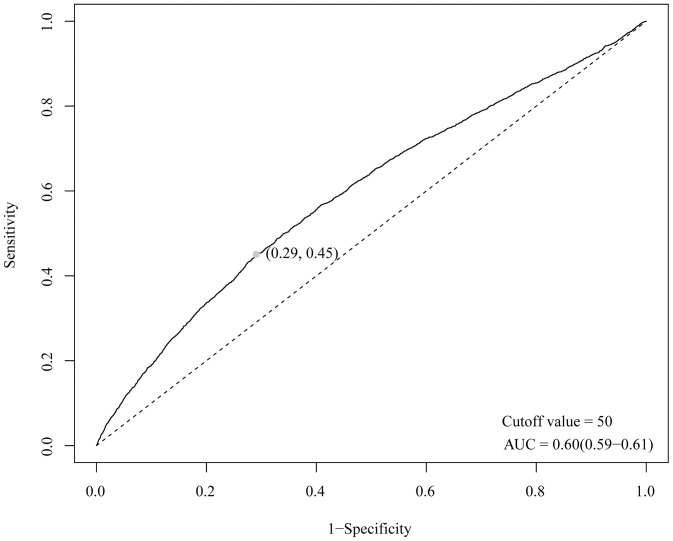
ROC curve for assessing the predictive effect of CUN-BAE for depression.

ROC analysis was utilized to examine the predictive validity of CUN-BAE for identifying individuals with depression (Fig 3). CUN-BAE predicted the AUC (95% CI) of 0.60 (0.59, 0.61), and the cutoff value is 50. CUN-BAE had a better predictive performance in identifying depression patients.

### 3.5. The mediating role of SIRI

**Fig 4:**
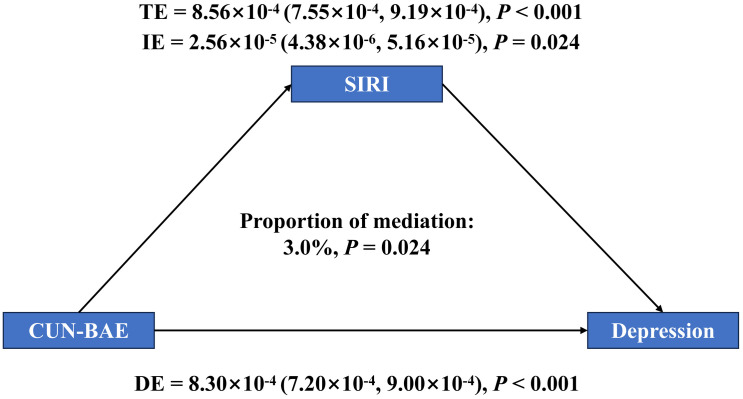
Mediation analysis of exploring the mediating effect of SIRI. Model was adjusted for age, gender, race, education, PIR, marital status, smoking status, drinking status, diabetes and hypertension.

Mediation modeling was conducted to evaluate the intermediary role of SIRI in the pathway linking CUN-BAE to depression (Fig 4). The TE of CUN-BAE on depression was 8.56 × 10 ⁻4 (95% CI: 7.55 × 10 ⁻4, 9.19 × 10 ⁻4, *P* < 0.001). The IE through SIRI was 2.56 × 10 ⁻5 (95% CI: 4.38 × 10 ⁻6, 5.16 × 10 ⁻5, *P* = 0.024), accounting for 3.0% of the total effect. The DE was 8.30 × 10⁻4 (95% CI: 7.20 × 10 ⁻4, 9.00 × 10 ⁻4, *P* < 0.001).

## 4. Discussion

Drawing on data from a large-scale cross-sectional cohort, our analysis identified a statistically significant positive relationship between CUN-BAE and depression. The application of the RCS regression model revealed a non-linear yet positive correlation between CUN-BAE and the presence of depression. The AUC value of the CUN-BAE index for diagnosis of depression was 0.60 (0.59, 0.61). The result of mediation effect illustrated that SIRI partially mediated the relationship between CUN-BAE and depression. Our research illustrated that higher levels of CUN-BAE are an independent risk factor for depression, and improving lipid metabolic status and inflammatory response might lessen the chance of developing depression.

Obesity and lipid deposition have been implicated as key contributors to depression. We constructed a large-scale cross-sectional investigation to systematically characterize the dose–response correlation between the CUN-BAE index and depression. We found that CUN-BAE was significantly and favorably correlated with depression, which is comparable to the results of earlier research. The Metabolic Score for Visceral Fat (METS-VF) is an important surrogate for exploring the level of visceral fat, and Liu et al. [28] suggested that the level of METS-VF was related to depression in individuals who were overweight or obese. Nong et al. [29] discovered a nonlinear correlation between depression and BMI. The CUN-BAE, as a comprehensive assessment of body fat that integrates age, gender, and BMI, is known as a more accurate evaluation of the body’s adiposity and distribution than BMI alone and is considered to be a more reliable marker for measuring obesity-related health risks [30,31]. On the one hand, elevated adiposity is linked to a chronic low-grade inflammatory state, which could lead to increased production of inflammatory substances, for example TNF-α, IL-1β, and CRP [32]. The latter, in turn, can trigger adrenocorticotropic hormone secretion, hypothalamus-adrenocorticotropic hormone axis activation, and cortisolism cascade response, which ultimately leads to neuronal apoptosis, dendritic atrophy, and reactive gliosis, which thus raises the chance of developing depression [33]. On the other hand, excessive body fat levels may trigger negative body imagery, social stigmatization, and decreased self-esteem, especially more pronounced in women. These psychosocial stressors may promote or exacerbate depression through pathways such as increased life stress and decreased social participation and support [34]. The above mechanisms may, in part, explain the significant positive link between CUN-BAE and the risk of depression.

In addition, the mediating effect results indicated that SIRI mediated the link between CUN-BAE and the risk of depression. The SIRI index, which is composed of neutrophil, monocyte, and lymphocyte counts, is acknowledged as a reliable indicator for assessing the state of inflammation in the organism. A substantial body of prior literature has highlighted the critical involvement of chronic low-grade inflammation in the pathogenesis of both obesity and depressive disorders [35–39]. We found that the SIRI index exhibited a significant mediating effect between CUN-BAE and the risk of depression, illustrating that systemic inflammation might be an important physiological mechanism linking body fat levels and the risk of depression. Excessive obesity can activate the immune system, which causes the release of pro-inflammatory mediators by immune cells [40], a process often associated with elevated SIRI values; and the sustained effects of inflammatory factors may interfere with neurotransmitter synthesis and metabolism, impacting mood regulation and brain function, raising the likelihood of depression [41]. However, it is worth emphasizing that although we observed a mediating effect of SIRI in the association between CUN-BAE and depression, the proportion of mediation is relatively small, indicating that SIRI represents only one of multiple potential mediating factors and cannot fully account for the relationship between CUN-BAE and depression.

There are some advantages to this study. First, we investigated the link between CUN-BAE and depression based on a comprehensive cross-sectional dataset with broad population representation. Second, we explored the mediating effect of the inflammatory index SIRI between CUN-BAE and depression, which provides new evidence to support the correlation between obesity and depression. The research does have a few limitations, though. First, we were unable to establish a causal correlation between the CUN-BAE index and depression because of the cross-sectional study design. Second, this study included a population from the United States only, and additional investigation is required to ascertain whether the results are transferable to other demographics and geographical areas. Third, although our findings suggest that SIRI may play a mediating role in the association between CUN-BAE and depression, the complex interrelationships among depression, inflammation, and adiposity mean that reverse or bidirectional associations among these three variables cannot be ruled out. Future longitudinal and mechanistic studies are warranted to further clarify their underlying relationships. Finally, although we adjusted for several covariates, we were unable to account for all potential factors, such as dietary supplement use, due to data limitations and complexity.

## 5. Conclusion

In conclusion, we illustrated that the CUN-BAE index was significantly and positively linked to the risk of depression, and SIRI had a mediating role between the two. This suggests that the level of CUN-BAE could be a potential indicator for assessing an individual’s risk of developing depression. Monitoring body fat and inflammation levels might be useful for early recognition of individuals potentially at risk for depression.

## Abbreviations

AUC: Area Under Curve
BMI: body mass index
CRP: C-reactive protein
CUN-BAE: Clínica Universidad de Navarra-Body Adiposity Estimator
DE: direct effect
IE: indirect effect
IL: interleukin
TNF-α: tumor necrosis factor-alpha
NHANES: National Health and Nutrition Examination Survey
PHQ-9: Patient Health Questionnaire-9
PIR: poverty income ratio
ROC: Receiver Operating Characteristic
SIRI: Systemic Inflammatory Response Index
TE: total effect.
